# Address

**Published:** 1883-05

**Authors:** Geo. R. Thomas

**Affiliations:** Detroit


### Address.

BY GEO. R. THOMAS, D.D.S., Detroit.

(Delivered at the Commencement of the Dental College of the University of Michigan, March 28th, 1883.)

MR. PRESIDENT, LADIES, AND GENTLEMEN:—We meet here to-day to witness the reception by these young men of their letters patent; that for which they have been toiling and hoping, and to the possession of which they have looked forward with eagerness and joyful anticipation. These are to be their passports into the realm of public favor and patronage; to them as yet an unexplored realm, peopled by many sunny fancies.

Gentlemen graduates, you have spent many months of study, in anticipation of this day; delving deep into books for necessary information, and teaching your hands to be skillful in the manipulation of those materials which are to aid you in the pursuit of your chosen profession.

Your studies have brought you to a stage in life where you break off from the instruction necessary to give you a position among professional men; you must now depend upon your own industry, and call into account all the faculties of which you are possessed, and of which you may even now be ignorant. There is no one who knows how much he is able to do or endure, till he makes an effort; or how many resources he may have in reserve, till he calls on them.

Dentistry is but young among the professions; it is less than a century since the subject was treated other than anatomically and philosophically. Its claims as a distinct branch of science have been recognized only about sixty years.

During the eighteenth century the practice of dentistry—all there was of it—appears to have been in the hands of silversmiths and jewelers. They made artificial teeth, after a fashion, and cleaned the natural teeth of those who should apply for such service; while the extraction of diseased teeth was relegated to barbers, and blacksmiths.

The primitive practitioners of dentistry had little thought of anything more than a separate practice; in fact, as late as 1828,

Noah Webster gives this definition of a dentist: "One whose occupation is to clean and extract teeth, or repair them when diseased." And if this definition embraced the whole curriculum of practice in that day, as we know it did, is it not gratifying to mark the change, and still more gratifying to predict the future? We can get an idea of the unappreciative condition of public opinion by referring to the address delivered at the first commencement of the first dental college in the world, by Prof. Thomas E. Bond, jr., M.D., in the year 1841, when there were but two graduates. He says: "You are aware that the Baltimore College of Dental Surgery went into operation under very discouraging circumstances. The difficulty of the undertaking is abundantly proved by the remarkable fact that notwithstanding the necessity of scientific education in dental surgery had long been felt, and the difficulty of procuring it bitterly lamented, no attempt had been made to afford collegiate opportunities to pupils in the art, until the faculty of the present school risked the adventure. It is unnecessary now to enter upon a minute detail of the causes which rendered success improbable. Suffice it to say that the projectors of the school had abundant reason to anticipate open hostility from many, and secret enmity from more. For this, however, they cared but little; but they feared that which is far worse than opposition; the entire indifference of the community to the result of the enterprise."

Having once fairly started, however, the impetus it acquired was something remarkable; and in 1858 it was estimated there were 4,000 dentists in the United States. But a very small proportion of these, however, were well instructed. Among those who were, there was a constant reaching out after something better. They were a company of ceaseless workers, invincible in courage, untiring in industry, who laid the foundations for the structure which now rears its head beside others of far greater antiquity, and of which we have no reason to be ashamed.

Their sons in the profession, who are still with us, have been carrying out and enlarging upon their ideas and teachings, keeping pace with the onward march of improvement, for they have ever kept in mind Goethe's motto, "Without haste, without rest."

With what affectionate regard do we speak the names of Professors Taft and Watt, Alport and Atkinson, the genial and never-to-be-forgotten pioneer, "Uncle Jerry" Robinson, and hosts of others whose deeds are cherished in our hearts. It is not often, however, that men are privileged to behold the results of their labors, and enjoy the fruition of their hopes. How many in the history of the world have "obtained a good report through faith, yet received not the promise," for we know that every age is very complacent as to its own achievements, and skeptical as to anything in advance.

Fortunately there is always a "Galileo" who will say: "It *does move.*"

When Mr. John Greenwood, in 1790, carved from ivory an entire set of teeth, with spiral spring attachments between upper and lower sets, for George Washington, it would have been quite as impossible to have predicted the possibilities of dentistry, and the benefits yet to be conferred upon suffering humanity, as it would have been in 1752, when Franklin was engaged with his key and kite, playing with the lightning, to have predicted all the wonderful electric possibilities that we now behold. Do you suppose that even *he* could have dreamed of the telephone, and the carrying of messages by submarine cable, even from Africa to the farthest verge of our own broad land, in a few minutes of time? Yet though he made a wondrous advance towards harnessing the lightning, it was nearly a century before Morse's mind conceived the idea of the electric telegraph. He, too, had prophetic glimpses of what should follow, when he visited England, a young man. In writing to his parents in New York, he says: "I only wish you had this letter *now*, to relieve your minds from anxiety; and that in an instant I could communicate my information; but 3,000 miles are not passed over in an instant, and we must wait for long weeks before we can hear from each other." And after having evidently finished the letter, he penned these words upon the back: "Longing for a telegraph, even in this letter." What must have been the gratitude of his heart, nearly twenty-five years later, when his daughter sent the first message over the wire to him, in these words: "What hath God wrought?"

The art of printing with movable types was not discovered till well along into the fifteenth century; and yet Gutenberg only gave expression to what must have been the earnest desire of those Chinese and Tartars who practiced the printing of books from engraved blocks, far back in the most remote periods; and although Gutenberg had brought to light a means by which human thought could be practically disseminated throughout the known world, it was more than a century before the people appreciated it enough to even recognize it in any way, which they did by publicly celebrating the event in Wittenberg, in 1540.

When Robert Fulton's little steamboat was laboring up the Hudson at the rate of four miles an hour, who would have believed that in less than three-quarters of a century there would be a line of fast steamers carrying people across the ocean in a little more than a week; or that they would be whirled over the land, across the valleys and through the mountains at the rate of nearly one hundred miles an hour? And if any one had ventured such a prophecy, the incredulous hearers would have voted the author a fanatic, and paid no attention to it, further than to have made the matter a subject of ridicule. It does seem a little strange to us in this day to reflect upon the fact, that within the memory of man, the Legislature of the great State of New York granted to Robert Fulton the exclusive right to the employment of all steam propulsion in that State, for the term of thirty years. And yet legislative bodies have not changed so very much, since those days, except in the means employed. Now, in great measure, the legislative steam reins are held by a few millionaires instead of by worthy inventors. Now legislative bodies are too apt to bow in submission to the mighty dollar instead of to scientific research. But in passing our gratuitous criticisms, and ofttimes anathemas, upon the actions of official bodies in their generous deeds toward the owners of the already plethoric purses, do we stop to consider that such stimulus may be necessary to the furtherance of undeveloped genius. As the skilled artisan is necessary to the development of the concealed brilliancy of the rough diamond? May it not be that Paul had reference to temporal, as well as spiritual things, when he said: "All things work together for good?"

Even in matters pertaining to the amelioration of suffering, and the prolongation of human life, the *public* and the *professions* have been lamentably slow in recognizing the claims of workers and discoverers. Jenner, the discoverer of vaccination, after experimenting until he could no longer doubt the result of its introduction, was met with such abuse and ridicule as would have daunted one less confident of his position; but though threatened with expulsion from the medical societies to which he belonged, if he continued to press his theory, he only pursued the theme more vigorously in another field. Not only did the doctors refuse to try his *process*, but brought him into contempt before the public; and his system was even denounced from the pulpit as diabolical.

Although Harvey did not suffer so severely as Jenner, still it was many years after he discovered the circulation of the blood before he received the credit he deserved.

The most eminent professors in England and on the Continent treated him with the hostility which all innovators have had to endure, though before his death they were forced to acknowledge the value of his discovery, and he had the satisfaction of witnessing the conversion of the most skeptical to the value of his opin ions, and the truth of his assertions.

In every age these master spirits have gloried in overcoming the difficulties which rose before them; and having leaped over one barrier, they pressed on with fresh courage to another. And what an example is furnished to you in this, young men. Your labor and application here have been long, and perhaps tedious; and you may have often thought, "well, it will soon be over, and then I can rest." True, you have reached a point where you may "call a halt," and take the needed rest, but it must needs be of short duration. The ever progressive movement of the time precludes long stops at any station; and he who imagines he has finished his studies will soon find he is left alone by the road-side, being out-distanced by his more ambitious fellows. How often you will hear men spoken of as "old fogies," "clear behind the times," because they are satisfied to rest with what they have already learned; they create nothing new themselves, and decry all advance movement in others. It will not do. And, gentlemen, when I suggest to you that your studies have but just begun, you may feel almost disheartened. A great man has said: "Nothing tends so much to the corruption of science as to suffer it to stagnate. These waters must be troubled before they can exert their virtues."

So the agitation of these depths of knowledge bring to the surface the riches contained therein. The labor and achievements of one man incite another to still greater efforts. Thus are we fellow helpers all. But you may say, "I have finished the course of study prescribed, have passed my examinations satisfactorily, I have here my certificate of qualification; what more is necessary?"

You have heretofore received your instruction from teachers and books; now you weigh anchor and cast off by yourselves; and everything depends on your skill and seamanship. Your boat is good and seaworthy. Do not hoist too much sail to begin with, but modestly keep near the shore till you see whether you are able to make a small venture; then, by degrees, launch out further and learn to weather the gales.

You have seen here some of the wonders revealed by the microscope, and have explored some of the mysteries of chemistry. Here, then, are two fields of almost boundless extent. Possess, if possible, a good microscope; and during the months to come, when you may have plenty of leisure, employ your time in a way that will result in some good. Lay up in store for the time to come.

Samuel Smiles has said: "Lost wealth may be replaced by industry; lost knowledge by study; lost health by temperance or medicine; but lost time is gone forever."

Many times troublesome cases will present themselves in your practice. Arm yourselves as best you may, and fortify your position by studying the experience of others; but verify the statements of your colleagues; do not accept them passively. "Prove all things, hold fast that which is good."

There is, as you probably by this time know—if you do not you soon will—a mooted question in the profession at present, namely: the causes of caries in the human teeth. There is no doubt but it will some day be decided, and some one will get the credit of it.

Then there will be the task of finding the remedy. If you espouse the chemical theory you have an abundant field of investigation before you, or if you take the other side and the germ theory, your microscope will be excellent company.

Do not be in haste to champion either side of the question, but look fairly at both. Apply your chemical tests thoroughly ; leave no experiment untried; and, on the other hand, apply your microscopes to every form of caries, and convince yourselves which is the true hypothesis on which to base your future action. We are all too apt to jump at conclusions, and take on trust the assertions of others, especially if they coincide with some preconceived notions of our own. Now, no *mere theory* is of much value, without accompanying proofs; but when we see the substantiation of the grounds upon which it is based, we are forced to accept it, even though some pet notion of ours has to be sacrificed in so doing. It is folly to plant ourselves in any position until we are sure of our foundation. Many a man will hold to a question, and argue in its favor when he *knows* he is wrong, rather than acknowledge his error. This, of course, is contrary to the spirit of progress and scientific enlightenment; but the old adage still holds good : "A man convinced against his will is of the same opinion still."

But some one may say, we are just starting out in the profession, and cannot afford a good microscope and the necessaries for scientific investigation. You would better far dispense with some articles of adornment in your offices; although I am far from belittling the art of beautifying and making pleasant and inviting. There are some matters in which you cannot afford to economize. "Expense, and great expense, may be an essential part in true economy, which is a distributive virtue, and consists not in saving, but in selection;" "this demands a discriminating judgment, and a firm sagacious mind."

The task of instructing others is also before you. You will be constantly thrown into contact with those who will need instruction as to the care of their teeth. And it must be "line upon line, and precept upon precept." Children will need those "words fitly spoken," which shall be to them in future, "apples of gold."

Do not deem it a light or trifling matter, this of teaching children to take care of those priceless organs. How many there are who would count no expense, or even pain, too great, if by these means they could replace that which they had lost by ignorance or neglect.

If you place yourselves before them in the guise of a kind instructor, and win their confidence, your influence will grow with their growth. Impress them with the idea that *they must* not only take daily care of their teeth, but at regular stated times come and let *you see* them, so you can be sure they are in perfect order, or if not, give them the attention they require.

It will not be presuming in you to do so. You are now going before the public *supposed* to be educated in your specialty. The *people*, as a whole, you will find, are deficient in your branch of education, and if you do not all in your power to elevate them to a higher plane, you are recreant to your trust. They are not supposed to know that if they leave their teeth till they begin to ache they are liable to lose them entirely. If your instruction is rightly given you are in no danger of appearing to force yourselves upon your patients, who will take your advice in the spirit in which you give it. And here, gentlemen, is a field for labor in which you should make your influence felt.

What *is* or *should be* the office of the progressive dentist of to day? He may possibly have satisfied his patient when he has performed the operation of *cleaning*, *extracting* and *repairing* the diseased teeth. But has he satisfied the demands of the profession to which he belongs? The demands of suffering humanity? The demands of his own desire to know what are the causes at work, to have made his services necessary, and to be able, if possible, to give advice that shall enable his patients to avert the necessity for so much reparative practice?

I have some time, and somewhere, seen it recorded of the people of a certain island, that there was by them entertained a religious superstition in regard to allowing their cats to retain their caudal appendages; the result of which was the amputation of the tails of all the kittens born upon the island for a number of generations, till eventually the people were spared the necessity of continuing that barbarous practice by the kittens coming into the world tailless.

Now, if the profession of dentistry, as a profession, was aiming to extract all the imperfect teeth that could be laid hold of, then we might predict for the distant future an edentulous race of human beings. But instead of this, the profession of dentistry has for its object--(I am not speaking of the ruthless tooth destroyers who infest all our large cities, and many small ones, and attempt to make the people believe, by a free use of printers' ink, that they are a part of the dental profession) I say, the dental profession has for its object, not only the preservation to the longest possible period of all the dental organs, but the enlightenment of the *people* (especially mothers), so that the children of coming generations may be as free from dental imperfections as possible.

But I hope, gentlemen, it is unnecessary (after all the training you have had in this institution) for me to call your attention to the necessity of the constant and persistent advice to parents, and to children who are to become parents, as to the necessity of obeying hygienic laws, the disobedience of which is just as much the cause of defective teeth as it is of disease and suffering of any other nature. Be not sparing of your advice as to diet, exercise and rest; tell the mother what will make good teeth for her children. The same kind of food that makes good teeth for the lower animals, will make good teeth for children as well. The young of the lower animals, during the formative state, get lime, silex, potash, phosphorus, and all the other elements that enter into good teeth, from the food taken by the mother, because she takes the food as nature has made it. And can the mother who lives principally on starch, butter and sugar, either of which contains scarcely a particle of lime, silex, potash, or phosphorus, expect the teeth of her offspring to be perfect, or even passably so? Nature performs no miracles.

Gentlemen, my advice to you is, place your ideal of dental achievement high up—*very high*, and then work to it; for if your ideal is low, your work will be correspondingly low.

Imagine, if you can, one, who twenty years ago, was in the foremost rank of the profession, having taken a "Rip Van

Winkle" sleep, awaking to-day to find himself in a well-equipped modern dental office. Think you he would feel at home? Scarcely would he know the use of a single instrument or appliance.

The turnkey has given way to the forceps; the hand-drill to the dental engine with all its equipments. The cumbrous and uncomfortable dental chair to the present marvel of elegance and comfort; the saliva-saturated napkins to the *priceless* elastic coffer dam; these, and many more within the space of about twenty years; and, yet, is it becoming for the profession, even in this day, to cry "Eureka?" Not yet! not yet! But, gentlemen, as you go forth into the world members of a noble and honorable profession, the friends of this institution will expect from you such conduct as will reflect credit upon your Alma Mater, and the profession to which you belong.

The dignity and honor of any profession is closely connected with its individual members, and the man who, by his vice or negligence, degrades his profession, is a traitor to his brethren, and a libeler of his class. The superiority to which your titular distinction entitles you will require continual diligence to maintain.

Desire earnestly the best things, and always be ready to take a step up. You will find that every worthy acquisition is a stepping-stone by which you will make a gradual ascent; and you know "there is always room at the top." You should be anxious not only to acquire information, but ready to impart it to others: that is, consider that any valuable hint which you may possess is not yours only, but belongs to the profession—to humanity. It is contrary to the spirit which should govern a liberal profession, to withhold any knowledge or discovery which may tend to the public good. It is every man's duty to add all he possibly can to the common stock of knowledge, and if each follows this rule, the expansion and progression of dental science is inevitable.

Never allow yourselves to be satisfied with a poor operation, and ever strive to fill the highest requirements; *then* you can expect a corresponding remuneration for your services; and if you sustain the dignity of this position, it will not fail to meet appreciation.

You will find, gentlemen, that a full practice of dentistry means *labor*—great *labor*—and weariness of mind and body, and ofttime spirit; and you will have need to cultivate that beautiful virtue, patience. Let it have its "perfect work," without which you are in no wise fitted to minister to delicate ladies and children, for whom your services are at best clothed with dread. The quick, sharp word, or impatient gesture, will add ten-fold to their discomfiture, and in nowise benefit yourself; whereas the gentle tone, and the kindly touch, will never fail to be appreciated. A kind and courteous demeanor is becoming in all, and is grateful to all. Not that politeness which consists merely in an observance of the coaventional rules of so-called "society," but that element which renders it impossible for you to do a coarse and rude thing; which is ever seeking the comfort and happiness of others; not alone of those whose wealth renders them desirable as patients, but to those whose only requital for your services will be the gratitude which they show. The physicians who visit the poor and minister to them in sickness gratuitously are true philanthropists; and there are many such. Their memory is beloved after they are gone; truly "their works do follow them." There is no reason why a dentist may not be as truly benevolent as a physician.

A dentist, to be sure, may not have as frequent opportunity for benevolence as a physician, but you will often find occasion for its exercise; and in a liberal profession its members should be governed by a wise charity which, like mercy, is twice blest. It blesses him that gives and him that takes. And, gentlemen, there is another subject which I would like to impress *deeply* upon your minds, viz.: your intercourse with brother dentists. The jealousies of professional men are so common that they have become proverbial. Now, there is no occasion for this; but on the contrary, every reason why you should be on the best of terms with men who are honorably pursuing the same profession with you. The world is wide, and there is plenty of room for you to grow and expand, both in knowledge and practice. You will be the gainer by a friendly intercourse with other dentists, not only outside their offices, but inside. Speak evil of none; if you can say no good of a man, say nothing at all, unless by keeping silent you do some one wrong. Do not fail to connect yourselves with the local Society wherever you may locate, and do your part to make it a working, progressive organization. Subscribe *freely* for the dental journals, and read them, too; do not get them for show, but let the experience of others guide you over some of the rough places you will encounter.

During the period in which the Olympian games were practiced in ancient Greece, the athletes, after a long time spent in training, presented themselves at specified times for a trial of speed and skill as well as strength.

This was to be a test of physical strength and endurance, without which their skill was of no avail. Their efforts were all to culminate in one day's exhibition, when, if they won the prize, they received the plaudits of the assembled multitude, and perhaps a crown of laurel leaves, which they considered ample reward for all their toil.

You gentlemen are just entering the arena for the long day of professional life; you must wrestle with many difficulties, and run the gauntlet of many adverse circumstances. But if you are men of mettle, those will be but the spur to your ambition, and urge you to greater efforts.

And, now, gentlemen, the eyes of the profession will be upon you. Honor yourselves, and in so doing honor this institution. And let us hope that through your lives you may be rewarded by the honor and gratitude of your fellow men, which will be a chaplet more enduring than gold or laurel; more to be sought after than empty applause, and which, in your riper years, will give you a satisfaction far higher than that obtained through physical endurance or prowess. Gentlemen, I bid you God speed.

